# The evolution of paternal care: a role for microbes?

**DOI:** 10.1098/rstb.2019.0599

**Published:** 2020-08-10

**Authors:** Yael Gurevich, Ohad Lewin-Epstein, Lilach Hadany

**Affiliations:** School of Plant Sciences and Food Security, Tel-Aviv University, Tel-Aviv 6997801, Israel

**Keywords:** microbiome, paternal care, mathematical model, extra-pair mating, sexual conflict, non-genetic inheritance

## Abstract

Paternal care, particularly in cases of uncertain paternity, carries significant costs. Extensive research, both theoretical and experimental, has explored the conditions in which paternal care behaviour would be favoured. Common explanations include an adjustment of care with uncertainty in paternity and limited accuracy in parentage assessment. Here, we propose a new explanation that microbes may play a role in the evolution of paternal care among their hosts. Using computational models, we demonstrate that microbes associated with increased paternal care could be favoured by natural selection. We find that microbe-induced paternal care could evolve under wider conditions than suggested by genetic models. Moreover, we show that microbe-induced paternal care is more likely to evolve when considering paternal care interactions that increase microbial transmission, such as feeding and grooming. Our results imply that factors affecting the composition of host microbiome may also alter paternal behaviour.

This article is part of the theme issue ‘The role of the microbiome in host evolution’.

## 1. Introduction

When would a father benefit more from caring for its offspring, rather than looking for additional mating opportunities? This question has been broadly addressed both theoretically and experimentally. Paternal care has been frequently observed among avian species [1] (approx. 85%), and also found in mammalian species [2] (approx. 5%), amphibians [3], and many species of fish [4]. Paternal care is most commonly observed alongside maternal care, while exclusive paternal care is rare [4,5]. A male may demonstrate care for its offspring with several types of interactions [4,5], such as feeding, grooming or guarding against predators. It can also provide spousal care for the female while she cares for the young [6,7].

A commonly proposed explanation for the prevalence of paternal care is borrowed from classical evolutionary theory [8]. This explanation suggests that paternal care would be favoured whenever the paternal contribution to offspring survival increases paternal fitness more than pursuing additional mating opportunities [9]. Nevertheless, studies relating paternal effort to the certainty of paternity have obtained mixed results [10,11]. Interestingly, paternal care is also observed in species where a sizable proportion (10–90%) of broods contain extra-pair offspring [12–14]. In some cases, males even knowingly care for unrelated young [15]. When a simple fitness trade-off falls short of justifying substantial paternal investment, alternative explanations have been suggested [16–19].

Here, we consider the potential role of microbes in host paternal care. The microbiome is a significant agent affecting host health and behaviour [20,21], through pathways such as the ‘gut–brain axis’. Numerous studies have demonstrated a possible association between microbes and social behaviour (reviewed in [20–22]), and certain species of the microbiome have been showed to alleviate symptoms of anxiety and depression [23] and improve social interactions [24]. Microbes are highly heritable, through gestation/incubation [25,26] or parental care [27–29]. Microbes can also be transmitted horizontally in a social setting [30], through interactions such as feeding, grooming and copulation [29]. The effect of microbes on host behaviour has given rise to the idea that host manipulation by microorganisms may be driven by natural selection on the microbes [22]. Selection could drive such an effect when the induced behaviour increases microbial fitness, for example, by increasing the rate of microbial transmission or proliferation [22], including host proliferation. Previous theoretical studies suggested that by encouraging host sociality [31] or altruistic behaviour [32], the microbes can help their own propagation.

We integrated the notion of microbe-associated behaviour into mathematical models for the evolution of paternal care. A family is a unit with a high probability of microbial transmission [33], since the members of the family partake in frequent and profound interactions. Caring for the young presents an excellent opportunity from a microbial perspective, since providing care both increases the odds of offspring survival [34] and establishes a higher transmission probability. Therefore, a microbial gene that is associated with host intra-family caring behaviour could be favoured by natural selection even when encouraging care towards genetically unrelated young individuals. The propagation of microbes carrying these genes may have contributed to the maintenance of paternal care even when paternity levels were rather low. Potential candidate microbes, such as *Lactobacillus* [35] and bifidobacteria [36], have been shown to impact mood or emotional behaviour and could be associated with paternal care.

## 2. Results

We examine two possible family structures. In every family, there is one female, who is mother to all offspring in the family. In the first family structure, all offspring are fathered by a single male, making them full siblings. In the second family type, we allow mixed paternity within the brood, while still maintaining a social pair structure.

### (a) Model I: broods of full siblings

In this model, males adopt one of two pure strategies, either paternal care or lack thereof. Offspring fitness is increased by paternal care [34], due to provision and protection from predation, by a factor of *s* > 0. The fitness of an offspring whose father does not provide paternal care is *ω_β_* = 1, while an offspring that receives paternal care has increased fitness *ω_α_* = 1 + *s*. Females mate with only one male. A male who provides paternal care can mate with one female and form a social pair. A male who does not provide paternal care can mate with more than one female and is limited only by female availability and receptivity [9]. The two types of males are subject to competition and sexual selection, wherein the caring males suffer a competitive disadvantage relative to the non-caring males (see [37] for discussion). The expected number of matings for both types of males is frequency-dependent [38], governed by the initial male composition in the mating pool. Only a fraction of the males of each type reach mating at all [39], thus the expected number of matings for a young male of the caring type is usually less than 1. Total male mating opportunities are bound by the Fisher condition [40], with a mean of 1. We assume that there is a cost to paternal care [8] and that the expected number of matings for a caring male decreases with paternal care [9].

First, we examine a population of non-caring males where the paternal behaviour is genetically driven. A rare mutant providing paternal care will have a higher fitness than a non-caring male when (at *p* = 0)

2.1 $$1+s>\frac{n_{\beta }}{n_{\alpha }},$$

where *n_β_* is the expected number of matings for a non-caring male, *n_α_* the expected number of matings for a caring male and *s* the increase in offspring fitness due to paternal care.

**Figure 1.**
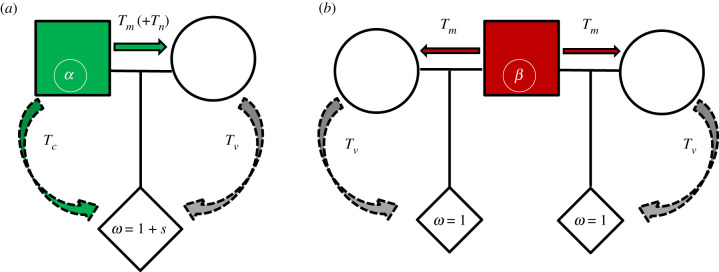
Model 1 (full siblings): illustration of microbe transmission pathways within the family: (*a*) where the father carries microbes of type *α*, inducing paternal care and (*b*) where the father carries microbes of type *β*, that have no effect on behaviour. Males carrying *β* do not care for the offspring and can be involved in *n* additional matings (illustrated is the case *n* = 1). *T_v_*, vertical transmission probability through maternal influence (prenatal and postnatal); *T_c_*, probability of transmission through paternal care; *T_m_*_,_ probability of male-to-female microbe transmission during mating; *T_n_*, probability of transmission through male-to-female allofeeding.

**Figure 2.**
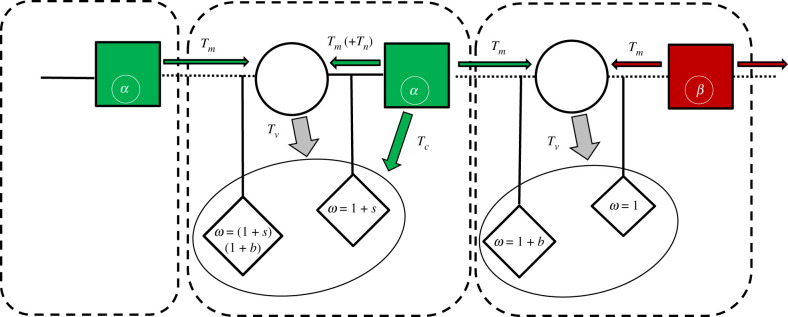
Model II (mixed brood): illustration of microbe transmission pathways within families with extra-pair mating. Males carrying microbes of type *β* do not care for the offspring, while males carrying microbes of type *α* care for the offspring in their social nest. All males and females engage in extra-pair mating. *T_v_*_,_ vertical transmission probability through maternal influence (prenatal and postnatal); *T_c_*, probability of transmission through paternal care; *T_m_*, probability of male-to-female transmission during mating; *T_n_*_,_ probability of transmission through male-to-female allofeeding. Offspring sired by an extra-pair mate are 1 + *b* times more fit than offspring sired by the social mate. Offspring that receive paternal care are 1 + *s* times more fit than offspring that do not receive it.

Now, we extend the model to include microbes as a reproductive unit that can affect paternal care behaviour. For simplicity, we neglect the effect of host genetic background in the microbe model and assume that host paternal behaviour is determined by its microbes. Let us consider microbes of type *α*, which are associated with paternal care behaviour, and microbes of type *β*, which have no effect on paternal care behaviour. Microbes can be transmitted to the offspring from the mother, with probability *T_v_*, or from the father, with probability *T_c_* when the father cares for the offspring. If parental microbes fail to establish in the offspring, it can adopt microbes horizontally by interacting with the general population [41], with probability determined by the population frequencies. In many species, allofeeding behaviour is more common from male to female than vice versa [42,43]. In our model, microbes can also be transmitted from the father to the mother during mating with probability *T_m_* and possibly through allofeeding of the mother, with probability *T_n_*. For simplicity, we assume that females carry the microbe, but it does not affect their behaviour. We assume that each host is inhabited by a single type of microbe at a given time. A transmission probability thus includes the probabilities that a microbe transmits to a new individual, establishes and replaces the resident microbe, encompassing the competition dynamics between different microbial strains. The transmission pathways and transmission probabilities of the two microbes are illustrated in figure 1. We consider a model where the mother cares for the offspring [44,45] and additionally can transmit microbes during gestation [26] and natally [25], so overall maternal transmission is higher than paternal transmission (*T_v_* > *T_c_*). We also assume that paternal care involves more interaction—and potential for microbe transmission—than a singular mating encounter (*T_c_* > *T_m_*). Since the probability of transmitting microbes during mating is asymmetric between the sexes, with a higher probability for male-to-female transmission [46], we neglect the probability of female-to-male transmission. We assume a delay in the effect of the microbes on behaviour and neglect the possibility of a male altering its paternal behaviour due to contracting different microbes at the mating stage. We initially assume that males have full paternity in their brood and relax that assumption later (figure 2).

We assume the following order of events within the reproductive process: transmission via mating occurs first, second is maternal transmission and finally transmission via paternal care, if it exists.

The condition for the evolution of microbe type *α* (see electronic supplementary material for full derivation) is given by

2.2 $$1+s>\frac{n_{\beta }}{n_{\alpha }}\cdot \left(\frac{T_{m}\cdot T_{v}}{T_{c}\;+\;T_{n}\cdot T_{v}\cdot (1\;-\;T_{c})}\right),$$

where *n_β_* is the expected number of matings for a non-caring male (at *p* = 0), *n_α_* the expected number of matings for a caring male (at *p* = 0), *s* the increase in offspring fitness due to paternal care, *T_v_* the vertical transmission probability through maternal care, *T_c_* the probability of transmission through paternal care, *T_m_* the probability of male-to-female microbe transmission during mating and *T_n_* the probability of transmission through male-to-female allofeeding.

**Figure 3.**
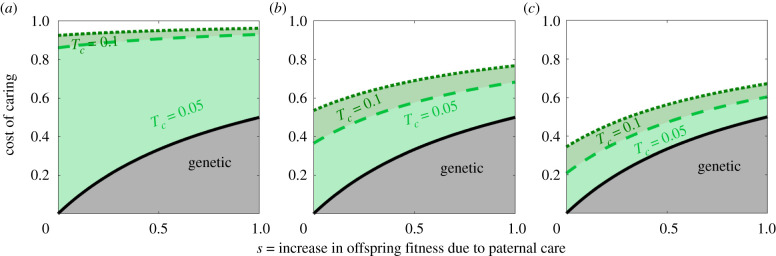
Model I (full siblings): microbes can expand the conditions for the evolution of paternal care. *T_c_*_,_ probability of microbe transmission from father to offspring through paternal care; *T_m_*, probability of transmission from male to female during mating. The area below each graph represents the conditions allowing paternal care to evolve in the population. A microbe associated with paternal care behaviour can widen the range of conditions where paternal care prevails, and the effect increases with the transmission probability of the paternal microbes to the offspring during care. Other parameters: *T_n_* = 0, *T_v_* = 0.8. (*a*) *T_m_* = 0.01, (*b*) *T_m_* = 0.1 and (*c*) *T_m_* = 0.2.

Figure 3 presents the range of fitness costs of caring imposed on the male that allows for the evolution of paternal care in the model. When *p* = 0, then the number of matings for a mutant of type *α* is $n_{\alpha }^{\;p=0}=\text{exp}(-\gamma_{c}\cdot s)$, while the number of matings for type *β* is $n_{\beta }^{\;p=0}=1$. We define the cost of caring, $C=n_{\beta }^{\;p=0}-n_{\alpha }^{\;p=0}=1-\text{exp}(-\gamma_{c}\cdot s)$, as the loss of mating success for a rare caring mutant of type *α*, where *γ_c_* is a factor governing the cost of caring. In the genetic case, the cost–benefit isocline is given by equation (2.1). The range of conditions where a gene for paternal care evolves is shown by the grey area (figure 3). The conditions where a microbe inducing paternal care evolves can be much wider, shown by the areas below the green lines. The range widens with *T_c_*, the probability of microbe transmission through paternal care, and narrows with *T_m_*, the transmission probability during mating (figure 3). Even when transmission through mating is larger than transmission through care, microbe-induced paternal care fares better than paternal care driven by a host gene (see electronic supplementary material, figure S1). This is in large part due to the maternal transmission of microbes to the offspring, the probability of which is assumed to be stronger than that of maternal genes (*T* = 0.5). However, reduced maternal transmission also allows microbe-induced paternal care to evolve quite easily (see electronic supplementary material, figure S2). Counterintuitively, the results also demonstrate that microbial genes inducing paternal care behaviour can evolve even in the paradoxical case where paternal ‘care’ decreases offspring fitness (see electronic supplementary material, figure S3).

Overall, if the ratio of transmission probability through paternal care to the probability through mating is sufficiently large, microbe-induced paternal care widens the range of conditions that allows for the evolution of paternal care behaviour. This is more significant when paternal care carries a substantial cost to the benefactor in terms of mating success or does not provide enough benefit to the beneficiary.

### (b) Model II: mixed brood

Now we consider a different social structure, where both males and females can engage in extra-pair mating, but offspring are brought up by social pairs [47–50]. Males of both types are guaranteed to form a social pair. Females mate with one extra-pair male besides their social mate, and a fraction *P_e_* of their brood are sired by the extra-pair mate. This factor is affected by mate guarding, sperm competition and cryptic female choice [51]. We assume that male mating success as an extra-pair sire reduces with paternal care of that male [9]. The more the father invests in its offspring, the fitter they will be, but the father may have fewer extra-pair progeny. The fitness of an offspring cared for by its social father is increased by a factor of 1 + *s*. The fitness of an extra-pair offspring is increased by a factor of 1 + *b*, due to direct or indirect benefits gained from extra-pair mating [52,53]. Note that this extra-pair offspring can still benefit from the care of its social father.

Let *ω_xyz_* be the fitness of an offspring with a social father of type *x*, a mother of type *y* and a biological father of type *z* (denoted by *ω_xy_* if the social father *x* is also the biological father). From our assumptions, *ω_αβα_* = *ω_αβ_*_*β*_ = *ω_ααα_* = *ω_ααβ_* = (1 + *b*) ∗ (1 + *s*), *ω_βαα_* = *ω_βαβ_* = *ω_ββα_* = *ω_βββ_* = (1 + *b*), while *ω_αa_* = *ω_αβ_* = (1 + *s*) and *ω_βa_* = *ω_ββ_* = 1.

We denote the expected number of extra-pair matings for a caring and non-caring male as *n_α_*, *n_β_*_,_ respectively. We define $n_{\alpha }=\exp(-\gamma_{c}\;*\;s\;*\;(1-p))$, where *γ_c_* is a factor governing the cost of caring, *s* is the increase in offspring fitness from paternal care, and *p* is the population frequency of *α*, the microbe associated with paternal care. Thus, when a rare mutant microbe inducing paternal care emerges in a population of males carrying microbes of type *β*, it will obtain $\exp(-\gamma_{c}\;*\;s)$ matings. On the other hand, if the population comprises only caring males, they will obtain, on average, one extra-pair mating opportunity.

We find the conditions for the evolution of an *α* host gene, coding for paternal care and similarly for the evolution of microbes of type *α*, inducing host paternal care (see electronic supplementary material, for mathematical derivations).

**Figure 4.**
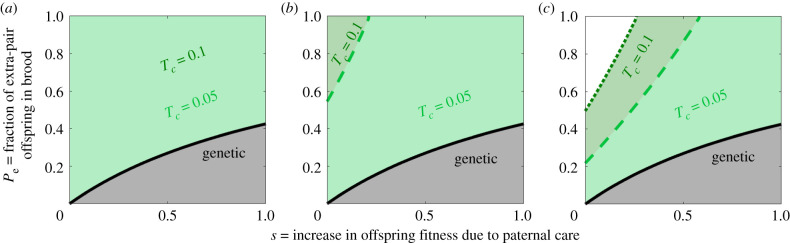
Model II (mixed brood): the evolution of paternal care in the face of extra-pair offspring in the brood. The figure represents the maximal fraction of extra-pair offspring in brood, *P_e_*, that allows for the evolution of paternal care induced by either genes or microbes. The solid lines represent the genetic case and the dashed and dotted lines represent the microbial cases. Generally, in the microbial case, paternal care evolves under wider conditions. The range narrows with an increase in extra-pair paternity in both the genetic and microbial cases. However, the effect is reduced when the transmission probability through paternal care (*T_c_*) is high. The different plots (*a*), (*b*) and (*c*) represent different values of transmission through mating (*T_m_*). In both the microbial and the genetic cases, as *s* increases, this allows for paternal care under higher degrees of extra-pair paternity. Other parameters: *b* = 0.5, *T_n_* = 0, *T_v_* = 0.8, *C* = 0.9; (*a*) *T_m_* = 0.01, (*b*) *T_m_* = 0.1 and (*c*) *T_m_* = 0.2.

Figure 4 shows the maximal fraction of extra-pair offspring in brood, *P_e_*, that allows for a gene or a microbe of type *α* (paternal care) to evolve. A high degree of extra-pair paternity in the population has a dual effect in the same direction. First, it allows for more opportunities to breed as an extra-pair sire. Males of type *β* have greater mating success as extra-pair sires than males of type *α*, thus could potentially gain more from the benefits to offspring stemming from extra-pair mating. We examine the effect of extra-pair mating benefits (*b*) in electronic supplementary material, figure S4. Second, it reduces the genetic relatedness of the social father to the offspring in its nest, and thus, the fitness benefits it receives from paternal care. This effect is stronger in the genetic case, since from a microbial perspective, paternal care for a genetically unrelated young individual contributes the same fitness benefits as for a genetically related one.

The increase in offspring fitness due to paternal care (*s*) affects the evolution of paternal care in an intricate manner, as both the benefits and the costs are increased with paternal investment. When the paternal contribution is sufficiently high, microbe-induced paternal care can evolve even when paternal care is subject to substantial costs (see electronic supplementary material, figure S5 for an example of diminished costs, where the evolution of microbe-induced paternal care is unlimited by paternity or paternal contribution).

**Figure 5.**
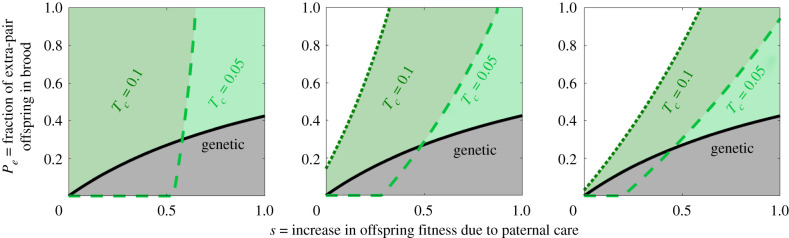
Model 2 (mixed brood): the evolution of paternal care in the face of extra-pair offspring in the brood, asymmetric transmission. Here, microbes of type *α* have a lower transmission probability than microbes of type *β* in mating interactions and through maternal transmission. When the fitness benefit of paternal care (*s*) or transmission probability through care (*T_c_*) are sufficiently high, microbe-induced paternal care allows for a wider range of costs than when driven by host genes. Otherwise, the substantially diminished success of microbes of type *α* via extra-pair behaviour hinders their evolution. The different plots represent different values of transmission through mating (*T_m_*). Other parameters: *b* = 0.5, *T_n_* = 0, *T_v_* = 0.8, *C* = 0.9; (*a*) *T_m_* = 0.01, (*b*) *T_m_* = 0.1 and (*c*) *T_m_* = 0.2 · $T_{\alpha }=0.9\cdot T_{\beta }$.

The dynamics between the two microbe types (*α* and *β*) are strongly affected by the ratio between transmission probability through paternal care (*T_c_*) and transmission probability through mating (*T_m_*). Generally, a higher *T_c_* allows for a wider range of conditions in which microbe-induced paternal care can evolve (see electronic supplementary material, figure S6 for extreme values of *T_m_*). Figure 5 demonstrates the asymmetric contagiousness case, when microbes of type *α* have a lower transmission probability than microbes of type *β* in mating interactions ($T_{m}^{\alpha }<T_{m}^{\beta })\;$and through maternal transmission ($T_{v}^{\alpha }<T_{v}^{\beta })$. In this case, when considering the extra-pair behaviour, microbes of type *α* have diminished success both due to the males' reduced mating opportunities and due to the lower transmission probability through mating. Hence, when the fraction of extra-pair offspring in the brood (*P_e_*) is high, the disparity in fitness increases in favour of microbes of type *β*. To counteract these costs and allow the care-inducing microbe to evolve, paternal care must provide a significant benefit. When the fitness benefit of paternal care (*s*) or transmission probability through care (*T_c_*) are sufficiently high, microbe-induced paternal care allows for a wider range of costs than when driven by host genes. We examined microbe-induced paternal care with transmission disadvantage for Model 1 as well (see electronic supplementary material, figure S7).

In conclusion, microbe-induced paternal care allows for the evolution of paternal care behaviour even in the face of the loss of paternity due to extra-pair mating, and when paternal investment results in significantly reduced mating success.

## 3. Discussion

In this work, we present a novel perspective into the benefits of paternal care under low levels of paternity and when paternal investment is costly. Our findings suggest that paternal care evolves more easily when induced by the microbes’ genes than by the host's genes. Our model predicts that microbe-induced paternal care would more easily evolve when parent–offspring interactions lead to a significant increase in offspring fitness, and when the probability of microbial transmission is high [41,54–57] (high *T_c_*, probability of paternal transmission of microbes via care, resulting from interactions corresponding to feeding, grooming). We demonstrate that microbe-induced paternal care can explain the maintenance of paternal care even under high levels of extra-pair mating, when the cost of caring is high, and even in some cases when the microbe inducing paternal care has a transmission disadvantage.

Previous work has discussed possible adaptive explanations for male care behaviour when paternity is uncertain, and care is costly [16]. Suggested benefits include acquisition of skills that produce future direct fitness benefits [58]; increased opportunities for successful parenthood in the following year [17]; and increased chances of mating [19] or territory acquisition [58]. Additionally, since female choice is a key factor in determining male reproductive success, sexual selection may act directly to favour paternal care [8,59,60]. It has also been suggested that males overestimate the likelihood of paternity [61,62], which helps preserve a stable level of paternal care [16,63,64]. However, when the cost of caring is high, and the expected level of paternity is low, selection is expected to favour more suspicious males that reduce their paternal investment with increased risk of extra-pair mating [16,64–68]. Nevertheless, in some organisms, paternal care has been observed even in these cases [13,14,69]. We propose that microbial evolutionary benefits could contribute to the maintenance of paternal care. We expect that microbe-induced paternal care could play a more significant role in circumstances where the classic fitness trade-off may fall short of explaining the observed degree of paternal investment, such as in adoption [15], as well as in species where extra-pair paternity is common [48].

Thus, microbe-induced paternal care could mediate some of the costs associated with female extra-pair mating behaviour. The benefits of extra-pair mating for females [50,52,70,71] may be in obtaining a higher quality or more compatible sire [70] and bet-hedging by increasing the genetic diversity of offspring [72]. Extra-pair mating can also result in significant costs to the female. The possible costs include loss of care by social mate [73], male sexual aggression [74], increased sibling competition [75,76] and the risk of contracting sexually transmitted pathogens [77]. As demonstrated by our results, microbe-induced care by the social mate prevails under a wider range of paternity levels in comparison to care driven by host genes. Within-brood aggression between half-siblings [75] could also be mitigated by microbes, since relatedness among the microbes of the sibling is expected to be significant even if their genetic relatedness is not high [78,79].

Our model can be extended in several ways. We examined two extremes: paternal care governed exclusively by host genes or exclusively by microbial genes. However, the evolution of paternal care is likely driven by selection on reproductive units in both levels, possibly leading to intermediate levels of paternal care between host and microbial optimum. Additionally, host manipulation is unlikely to have arisen by a single mutation and more plausibly would evolve gradually through small cumulative changes. It is possible to consider that when host genes and microbial genes experience conflicting selective pressures, selection on the host would drive the evolution of resistance genes to the microbial influence. In this case, we expect the host–microbe coevolution to generate oscillatory rock–paper–scissors evolutionary dynamics, that can allow the long-term maintenance of paternal care. Similar dynamics have been suggested by some of us with respect to microbe-induced cooperation and host resistance [80]. It should also be noted that host manipulation does not have to require novel complex behaviour [81] but may more commonly take place through influencing pre-existing host behaviours [82,83]. Another extension would be allowing more female strategies. We assumed a constant level of maternal care. Yet, studies show that females may reduce their care if the male provides sufficiently intensive care or increase their care to compensate for lack of male care [84–86]. This behaviour lowers the return on paternal investment in terms of benefits to offspring fitness, effectively increasing the cost/benefit ratio. We may also consider microbial genes that contribute to the maintenance of maternal care. We predict that in many cases, microbial evolutionary interests would be to promote high levels of maternal investment, as the offspring is very likely to carry microbes of the same type as his mother, especially in cases where paternal involvement is meagre or lacking.

Our model joins the rank of previous models concerning the role of different non-genetic elements in the evolution of social traits [32,87–89]. Recent evidence suggests that microbes hold a significant role in shaping host evolution [21,32,90,91]. However, it is worth noting that the assumptions presented here are not limited to the microbiome and apply to any class of non-genetic elements that are capable of both vertical and horizontal/oblique [92] transfer and of influencing complex behavioural phenotypes. Examples of such elements may include epigenetic states [93,94] and culture [87,95–98]. Paternal care is a unique case of cooperation. The general outline of our model can also be applied to cooperation among genetic relatives with varying degrees of relatedness, cooperative breeding and eusociality [58,99].

Our theoretical results suggest a new evolutionary explanation, involving microbial regulation, to paternal care in the face of significant costs. Our results call for empirical testing of our predictions: that microbes are involved in the regulation of paternal behaviour and that factors that affect the composition of the host microbiome dramatically (e.g. antibiotics [100,101]) may also alter paternal behaviour.

## Supplementary Material

Supplementary material

## Supplementary Material

Supplementary figures

## Data accessibility

This article has no additional data.

## References

[1] Cockburn A 2006 Prevalence of different modes of parental care in birds. Proc. R. Soc. B 273, 1375–1383. (10.1098/rspb.2005.3458) PMC1560291 16777726

[2] Kleiman D, Malcolm J 1981 The evolution of male parental investment in mammals. In Parental care in mammals (eds Gubernick DJ, Klopier PH), pp. 347–387. New York, NY: Plenum Publishing.

[3] Brown JL, Morales V, Summers K 2010 A key ecological trait drove the evolution of biparental care and monogamy in an amphibian. Am. Nat. 175, 436–446. (10.1086/650727) 20180700

[4] Ridley M 1978 Paternal care. Anim. Behav. 26, 904–932 . (10.1016/0003-3472(78)90156-2)

[5] Clutton-Brock TH 1991 The evolution of parental care. Princeton, NJ: Princeton University Press.

[6] Shellman-Reeve J 1990 Dynamics of biparental care in the dampwood termite, *Zootermopsis nevadensis* (Hagen): response to nitrogen availability. Behav. Ecol. Sociobiol. 26, 389–397. (10.1007/BF00170895)

[7] Freeman H 1983 Behavior in adult pairs of captive snow leopards (*Panthera uncia*). Zoo Biol. 2, 1–22. (10.1002/zoo.1430020102)

[8] Trivers RL 1972 Parental investment and sexual selection. In Sexual selection and the descent of man (ed. Campbell BG), pp. 136–179. Piscataway, NJ: Aldine Transaction.

[9] Magrath MJL, Komdeur J 2003 Is male care compromised by additional mating opportunity? Trends Ecol. Evol. 18, 424–430. (10.1016/S0169-5347(03)00124-1)

[10] Sheldon BC 2002 Relating paternity to paternal care. Phil. Trans. R. Soc. Lond. B 357, 341–350. (10.1098/rstb.2001.0931) 11958702 PMC1692948

[11] Møller AP, Birkhead TR 1993 Certainty of paternity covaries with paternal care in birds. Behav. Ecol. Sociobiol. 33, 261–268. (10.1007/BF02027123)

[12] Brouwer L, Griffith SC 2019 Extra-pair paternity in birds. Mol. Ecol. 28, 4864–4882. (10.1111/mec.15259) 31587397 PMC6899757

[13] Bouwman KM, Lessells CM, Komdeur J 2005 Male reed buntings do not adjust parental effort in relation to extrapair paternity. Behav. Ecol. 16, 499–506. (10.1093/beheco/ari021)

[14] Kamel SJ, Grosberg RK 2012 Exclusive male care despite extreme female promiscuity and low paternity in a marine snail. Ecol. Lett. 15, 1167–1173. (10.1111/j.1461-0248.2012.01841.x) 22834645

[15] Riedman ML 1982 The evolution of alloparental care and adoption in mammals and birds. Q. Rev. Biol. 57, 405–435. (10.1086/412936)

[16] Griffin AS, Alonzo SH, Cornwallis CK 2013 Why do cuckolded males provide paternal care? PLoS Biol. 11, e1001520 (10.1371/journal.pbio.1001520) 23555193 PMC3608547

[17] Rosenbaum S, Vigilant L, Kuzawa CW, Stoinski TS 2018 Caring for infants is associated with increased reproductive success for male mountain gorillas. Sci. Rep. 8, 1–8. (10.1038/s41598-018-33380-4) 30323256 PMC6189178

[18] Wisenden BD 1999 Alloparental care in fishes. Rev. Fish Biol. Fish. 9, 45–70. (10.1023/A:1008865801329)

[19] Kerhoas D, Kulik L, Perwitasari-Farajallah D, Agil M, Engelhardt A, Widdig A 2016 Mother-male bond, but not paternity, influences male-infant affiliation in wild crested macaques. Behav. Ecol. Sociobiol. 70, 1117–1130. (10.1007/s00265-016-2116-0) 27478299 PMC4954837

[20] Cryan JF, Dinan TG 2012 Mind-altering microorganisms: the impact of the gut microbiota on brain and behaviour. Nat. Rev. Neurosci. 13, 701–712. (10.1038/nrn3346) 22968153

[21] Vuong HE, Yano JM, Fung TC, Hsiao EY 2017 The microbiome and host behavior. Annu. Rev. Neurosci. 40, 21–49. (10.1146/annurev-neuro-072116-031347) 28301775 PMC6661159

[22] Sherwin E, Bordenstein SR, Quinn JL, Dinan TG, Cryan JF 2019 Microbiota and the social brain. Science 366, eaar2016 (10.1126/science.aar2016) 31672864

[23] Foster JA, McVey Neufeld K-A 2013 Gut–brain axis: how the microbiome influences anxiety and depression. Trends Neurosci. 36, 305–312. (10.1016/j.tins.2013.01.005) 23384445

[24] Bharwani A, Mian MF, Surette MG, Bienenstock J, Forsythe P 2017 Oral treatment with *Lactobacillus rhamnosus* attenuates behavioural deficits and immune changes in chronic social stress. BMC Med. 15, 1–14. (10.1186/s12916-016-0771-7) 28073366 PMC5225647

[25] Mändar R, Mikelsaar M 1996 Transmission of mother's microflora to the newborn at birth. Neonatology 69, 30–35. (10.1159/000244275) 8777246

[26] Funkhouser LJ, Bordenstein SR 2013 Mom knows best: the universality of maternal microbial transmission. PLoS Biol. 11, e1001631 (10.1371/journal.pbio.1001631) 23976878 PMC3747981

[27] Banning JL, Weddle AL, Wahl Iii GW, Simon MA, Lauer A, Walters RL, Harris RN 2008 Antifungal skin bacteria, embryonic survival, and communal nesting in four-toed salamanders, *Hemidactylium scutatum*. Oecologia 156, 423–429. (10.1007/s00442-008-1002-5) 18335251

[28] Gunderson AR, Forsyth MH, Swaddle JP 2009 Evidence that plumage bacteria influence feather coloration and body condition of eastern bluebirds *Sialia sialis*. J. Avian Biol. 40, 440–447. (10.1111/j.1600-048x.2008.04650.x)

[29] Kulkarni S, Heeb P 2007 Social and sexual behaviours aid transmission of bacteria in birds. Behav. Processes. 74, 88–92. (10.1016/j.beproc.2006.10.005) 17118574

[30] Koch H, Schmid-Hempel P 2011 Socially transmitted gut microbiota protect bumble bees against an intestinal parasite. Proc. Natl Acad. Sci. USA 108, 19 288–19 292. (10.1073/pnas.1110474108) PMC3228419 22084077

[31] Stilling RM, Bordenstein SR, Dinan TG, Cryan JF 2014 Friends with social benefits: host-microbe interactions as a driver of brain evolution and development? Front. Cell Infect. Microbiol. 4, 147 (10.3389/fcimb.2014.00147) 25401092 PMC4212686

[32] Lewin-Epstein O, Aharonov R, Hadany L 2017 Microbes can help explain the evolution of host altruism. Nat. Commun. 8, 1–7. (10.1038/ncomms14040) 28079112 PMC5241693

[33] Rothschild D et al. 2018 Environment dominates over host genetics in shaping human gut microbiota. Nature, 555, 210–215. (10.1038/nature25973) 29489753

[34] Kölliker M 2012 The evolution of parental care. Oxford, UK: Oxford University Press.

[35] Bravo JA, Forsythe P, Chew MV, Escaravage E, Savignac HM, Dinan TG, Bienenstock J, Cryan JF 2011 Ingestion of *Lactobacillus* strain regulates emotional behavior and central GABA receptor expression in a mouse via the vagus nerve. Proc. Natl Acad. Sci. USA 108, 16 050–16 055. (10.1073/pnas.1102999108) PMC3179073 21876150

[36] Schmidt C 2015 Mental health: thinking from the gut. Nature 518, S12–S15. (10.1038/518S13a) 25715275

[37] Stiver KA, Alonzo SH 2009 Parental and mating effort: is there necessarily a trade-off? Ethology 115, 1101–1126. (10.1111/j.1439-0310.2009.01707.x)

[38] Kokko H, Jennions MD 2008 Parental investment, sexual selection and sex ratios. J. Evol. Biol. 21, 919–948. (10.1111/j.1420-9101.2008.01540.x) 18462318

[39] Ahnesjö I, Kvarnemo C, Merilaita S 2001 Using potential reproductive rates to predict mating competition among individuals qualified to mate. Behav. Ecol. 12, 397–401. (10.1093/beheco/12.4.397)

[40] Jennions MD, Fromhage L 2017 Not all sex ratios are equal: the Fisher condition, parental care and sexual selection. Phil. Trans. R. Soc. B 372, 20160312 (10.1098/rstb.2016.0312) 28760755 PMC5540854

[41] Tung J et al. 2015 Social networks predict gut microbiome composition in wild baboons. eLife 4, e05224 (10.7554/eLife.05224) PMC4379495 25774601

[42] Van Noordwijk MA, Van Schaik CP 2009 Intersexual food transfer among orangutans: do females test males for coercive tendency? Behav. Ecol. Sociobiol. 63, 883–890. (10.1007/s00265-009-0728-3)

[43] Poiani A 1992 Feeding of the female breeder by male helpers in the bell miner *Manorina melanophrys*. Emu 92, 233–237. (10.1071/mu9920233)

[44] Smiseth PT, Moore AJ 2004 Behavioral dynamics between caring males and females in a beetle with facultative biparental care. Behav. Ecol. 15, 621–628. (10.1093/beheco/arh053)

[45] Hunt J, Simmons LW 2002 Behavioural dynamics of biparental care in the dung beetle *Onthophagus taurus*. Anim. Behav. 64, 65–75. (10.1006/anbe.2002.3036)

[46] White J, Mirleau P, Danchin E, Mulard H, Hatch SA, Heeb P, Wagner RH 2010 Sexually transmitted bacteria affect female cloacal assemblages in a wild bird. Ecol. Lett. 13, 1515–1524. (10.1111/j.1461-0248.2010.01542.x) 20961376 PMC3772342

[47] Westneat DF, Sherman PW, Morton ML 1990 The ecology and evolution of extra-pair copulations in birds. Curr. Ornithol. 7, 331–369.

[48] Petrie M, Kempenaers B 1998 Extra-pair paternity in birds: explaining variation between species and populations. Trends Ecol. Evol. 13, 52–57. (10.1016/S0169-5347(97)01232-9) 21238200

[49] Griffith SC, Owens IPF, Thuman KA 2002 Extra pair paternity in birds: a review of interspecific variation and adaptive function. Mol. Ecol. 11, 2195–2212. (10.1046/j.1365-294X.2002.01613.x) 12406233

[50] Westneat DF, Stewart IRK 2003 Extra-pair paternity in birds: causes, correlates and conflict. Annu. Rev. Ecol. Evol. Syst. 34, 365–396. (10.1146/annurev.ecolsys.34.011802.132439)

[51] Eberhard WG 1996 Female control: sexual selection by cryptic female choice. Princeton, NJ: Princeton University Press.

[52] Forstmeier W, Nakagawa S, Griffith SC, Kempenaers B 2014 Female extra-pair mating: adaptation or genetic constraint? Trends Ecol. Evol. 29, 456–464. (10.1016/j.tree.2014.05.005) 24909948

[53] Jennions MD, Petrie M 2000 Why do females mate multiply? A review of the genetic benefits. Biol. Rev. Camb. Philos. Soc. 75, 21–64. (10.1017/S0006323199005423) 10740892

[54] Ezenwa VO, Ghai RR, McKay AF, Williams AE 2016 Group living and pathogen infection revisited. Curr. Opin. Behav. Sci. 12, 66–72. (10.1016/j.cobeha.2016.09.006)

[55] Moeller AH, Foerster S, Wilson ML, Pusey AE, Hahn BH, Ochman H 2016 Social behavior shapes the chimpanzee pan-microbiome. Sci. Adv. 2, e1500997 (10.1126/sciadv.1500997) 26824072 PMC4730854

[56] Song SJ et al. 2013 Cohabiting family members share microbiota with one another and with their dogs. eLife 2, 458 (10.7554/eLife.00458) PMC3628085 23599893

[57] VanderWaal KL, Atwill ER, Isbell LA, McCowan B 2014 Linking social and pathogen transmission networks using microbial genetics in giraffe (*Giraffa camelopardalis*). J. Anim. Ecol. 83, 406–414. (10.1111/1365-2656.12137) 24117416

[58] Cockburn A 1998 Evolution of helping behavior in cooperatively breeding birds. Annu. Rev. Ecol. Syst. 29, 141–177. (10.1146/annurev.ecolsys.29.1.141)

[59] Kokko H, Brooks R, Jennions MD, Morley J 2003 The evolution of mate choice and mating biases. Proc. R. Soc. Lond. B 270, 653–664. (10.1098/rspb.2002.2235) PMC1691281 12769467

[60] Moller AP 2000 Male parental care, female reproductive success, and extrapair paternity. Behav. Ecol. 11, 161–168. (10.1093/beheco/11.2.161)

[61] Neff BD 2003 Decisions about parental care in response to perceived paternity. Nature 422, 716–719. (10.1038/nature01528) 12700761

[62] Lissåker M, Svensson O 2008 Cannibalize or care? The role of perceived paternity in the sand goby, *Pomatoschistus minutus*. Behav. Ecol. Sociobiol. 62, 1467–1475. (10.1007/s00265-008-0576-6)

[63] Westneat DF, Sherman PW 1993 Parentage and the evolution of paternal care. Behav. Ecol. 4, 66–77. (10.1093/beheco/4.1.66)

[64] Kokko H 1998 Should advertising parental care be honest? Proc. R. Soc. Lond. B 265, 1871–1878. (10.1098/rspb.1998.0515)

[65] Manica A 2004 Parental fish change their cannibalistic behaviour in response to the cost-to-benefit ratio of parental care. Anim. Behav. 67, 1015–1021. (10.1016/j.anbehav.2003.09.011)

[66] Houston AI, McNamara JM 2002 A self-consistent approach to paternity and parental effort. Phil. Trans. R. Soc. Lond. B 357, 351–362. (10.1098/rstb.2001.0925) 11958703 PMC1692954

[67] Hamilton WD 1964 The genetical evolution of social behaviour. II. J. Theor. Biol. 7, 17–52. (10.1016/0022-5193(64)90039-6) 5875340

[68] Houston AI 1995 Parental effort and paternity. Anim. Behav. 50, 1635–1644. (10.1016/0003-3472(95)80017-4)

[69] Bose APH, Houpt N, Rawlins M, Miller JS, Juanes F, Balshine S 2020 Indirect cue of paternity uncertainty does not affect nest site selection or parental care in a Pacific toadfish. Behav. Ecol. Sociobiol. 74, 1–10. (10.1007/s00265-019-2778-5)

[70] Foerster K, Delhey K, Johnsen A, Lifjeld JT, Kempenaers B 2003 Females increase offspring heterozygosity and fitness through extra-pair matings. Nature 425, 714–717. (10.1038/nature01969) 14562103

[71] Kempenaers B, Dhondt AA 1993 Why do females engage in extra-pair copulations? A review of hypotheses and their predictions. Belg. J. Zool. 123, 93–103.

[72] Fox CW, Rauter CM 2003 Bet-hedging and the evolution of multiple mating. Evolut. Ecol. Res. 5, 273–286.

[73] Arnqvist G, Kirkpatrick M 2005 The evolution of infidelity in socially monogamous passerines: the strength of direct and indirect selection on extrapair copulation behavior in females. Am. Nat. 165(Suppl. 5), S26–S37. (10.1086/429350) 15795859

[74] Valera F, Hoi H, Krištín A 2003 Male shrikes punish unfaithful females. Behav. Ecol. 14, 403–408. (10.1093/beheco/14.3.403)

[75] Briskie JV, Naugler CT, Leech SM 1994 Begging intensity of nestling birds varies with sibling relatedness. Proc. R. Soc. Lond. B 258, 73–78. (10.1098/rspb.1994.0144)

[76] Brante A, Fernández M, Viard F 2013 Non-random sibling cannibalism in the marine gastropod *Crepidula coquimbensis*. PLoS ONE 8, e67050 (10.1371/journal.pone.0067050) 23805291 PMC3689673

[77] Sheldon BC 1993 Sexually transmitted disease in birds: occurrence and evolutionary significance. Phil. Trans. R. Soc. Lond. B 339, 491–497. (10.1098/rstb.1993.0044) 8098875

[78] Mosites E et al. 2017 Microbiome sharing between children, livestock and household surfaces in western Kenya. PLoS ONE 12, e0171017 (10.1371/journal.pone.0171017) 28152044 PMC5289499

[79] Brito IL et al. 2019 Transmission of human-associated microbiota along family and social networks. Nat. Microbiol. 4, 964–971. (10.1038/s41564-019-0409-6) 30911128 PMC7450247

[80] Lewin-Epstein O, Hadany L 2020 Host–microbiome coevolution can promote cooperation in a rock–paper–scissors dynamics. Proc. R. Soc. B 287, 20192754 (10.1098/rspb.2019.2754) PMC7031668 32075531

[81] Hughes DP, Andersen SB, Hywel-Jones NL, Himaman W, Billen J, Boomsma JJ 2011 Behavioral mechanisms and morphological symptoms of zombie ants dying from fungal infection. BMC Ecol. 11, 13 (10.1186/1472-6785-11-13) 21554670 PMC3118224

[82] McCurdy DG 1999 Evidence that the parasitic nematode *Skrjabinoclava* manipulates host *Corophium* behavior to increase transmission to the sandpiper, *Calidris pusilla*. Behav. Ecol. 10, 351–357. (10.1093/beheco/10.4.351)

[83] Alcock J, Maley CC, Aktipis CA 2014 Is eating behavior manipulated by the gastrointestinal microbiota? Evolutionary pressures and potential mechanisms. Bioessays 36, 940–949. (10.1002/bies.201400071) 25103109 PMC4270213

[84] Kokko H 1999 Cuckoldry and the stability of biparental care. Ecol. Lett. 2, 247–255. (10.1046/j.1461-0248.1999.00075.x)

[85] Houston AI, Székely T, McNamara JM 2005 Conflict between parents over care. Trends Ecol. Evol. 20, 33–38. (10.1016/j.tree.2004.10.008) 16701338

[86] Harrison F, Barta Z, Cuthill I, Szekely T 2009 How is sexual conflict over parental care resolved? A meta-analysis. J. Evol. Biol. 22, 1800–1812. (10.1111/j.1420-9101.2009.01792.x) 19583699

[87] Feldman MW, Cavalli-Sforza LL, Peck JR 1985 Gene-culture coevolution: models for the evolution of altruism with cultural transmission. Proc. Natl Acad. Sci. USA 82, 5814–5818. (10.1073/pnas.82.17.5814) 3862097 PMC390643

[88] Bonduriansky R, Day T 2018 Extended heredity: a new understanding of inheritance and evolution. Princeton, NJ: Princeton University Press.

[89] Bonduriansky R, Day T 2013 Nongenetic inheritance and the evolution of costly female preference. J. Evol. Biol. 26, 76–87. (10.1111/jeb.12028) 23163399

[90] Morimoto J, Simpson SJ, Ponton F 2017 Direct and trans-generational effects of male and female gut microbiota in *Drosophila melanogaster*. Biol. Lett. 13, 20160966 (10.1098/rsbl.2016.0966) 28724687 PMC5543016

[91] Ezenwa VO, Gerardo NM, Inouye DW, Medina M, Xavier JB 2012 Animal behavior and the microbiome. Science 338, 198–199. (10.1126/science.1227412) 23066064

[92] Ram Y, Liberman U, Feldman MW 2019 Vertical and oblique cultural transmission fluctuating in time and in space. Theor. Popul. Biol. 125, 11–19. (10.1016/j.tpb.2018.11.001) 30465795

[93] Danchin É, Charmantier A, Champagne FA, Mesoudi A, Pujol B, Blanchet S 2011 Beyond DNA: integrating inclusive inheritance into an extended theory of evolution. Nat. Rev. Genet. 12, 475–486. (10.1038/nrg3028) 21681209

[94] Jablonka E, Lamb MJ, Zeligowski A 2014 Evolution in four dimensions, revised edn Cambridge, MA: MIT Press.

[95] Bartsch C, Weiss M, Kipper S 2015 Multiple song features are related to paternal effort in common nightingales. BMC Evol. Biol. 15, 1–8. (10.1186/s12862-015-0390-5) 26084455 PMC4471916

[96] Murray CM, Gilby IC, Mane SV, Pusey AE 2008 Adult male chimpanzees inherit maternal ranging patterns. Curr. Biol. 18, 20–24. (10.1016/j.cub.2007.11.044) 18158245

[97] Cavalli-Sforza LL, Feldman M 1981 Cultural transmission and evolution: a quantitative approach, Princeton, NJ: Princeton University Press. 7300842

[98] Rushton JP, Littlefield CH, Lumsden CJ 1986 Gene-culture coevolution of complex social behavior: human altruism and mate choice. Proc. Natl Acad. Sci. USA 83, 7340–7343. (10.1073/pnas.83.19.7340) 3463973 PMC386712

[99] Clutton-Brock T 2002 Breeding together: kin selection and mutualism in cooperative vertebrates. Science 296, 69–72. (10.1126/science.296.5565.69) 11935014

[100] Jakobsson HE, Jernberg C, Andersson AF, Sjölund-Karlsson M, Jansson JK, Engstrand L 2010 Short-term antibiotic treatment has differing long-term impacts on the human throat and gut microbiome. PLoS ONE 5, e9836 (10.1371/journal.pone.0009836) 20352091 PMC2844414

[101] Manichanh C, Reeder J, Gibert P, Varela E, Llopis M, Antolin M, Guigo R, Knight R, Guarner F 2010 Reshaping the gut microbiome with bacterial transplantation and antibiotic intake. Genome Res. 20, 1411–1419. (10.1101/gr.107987.110) 20736229 PMC2945190

